# Wheel Diameter Affects Vibration Transmission but Not Performance During Uphill and Downhill Mountain Biking over Rough Terrain

**DOI:** 10.3390/mps9040118

**Published:** 2026-08-14

**Authors:** Enrique Moreno-Manas, Salvador Llana-Belloch, Gonzalo Monfort-Torres, Xavier García-Massó

**Affiliations:** 1 Physical Education and Sports Department, University of Valencia, 46010 Valencia, Spain; enrique.moreno-manas@uv.es (E.M.-M.); gonzalo.monfort-torres@uv.es (G.M.-T.); 2 Human Movement Analysis Group, Department of Teaching Physical Education, Arts and Music, University of Valencia, 46022 Valencia, Spain; xavier.garcia@uv.es

**Keywords:** mountain biking, wheel size, bicycle evolution, cycling biomechanics, vibration transmission

## Abstract

Mountain biking over rough terrain exposes riders to vibrations that may affect comfort, health, bicycle control, and performance. This study analyzed the effect of wheel diameter on vibration transmission and performance during a short uphill and downhill test over rocky terrain. Forty-nine highly trained male mountain bikers completed repeated trials using two equivalent hardtail mountain bikes with 26- and 29-inch wheels. Vibrations were recorded with eight triaxial accelerometers placed on the bicycle and rider, and performance was assessed using an electronic photocell timing system. Both wheel sizes showed a similar vibration pattern, with lower root mean square (RMS) acceleration values at the helmet and coccyx and higher values at the wrists and ankles. However, wheel diameter significantly influenced vibration transmission. During the uphill test, the 26-inch bicycle produced higher accelerations at the coccyx and rear hub, whereas during the downhill test, the 29-inch bicycle transmitted greater vibrations to the rider’s wrists and ankles. No significant differences were observed between wheel sizes in the time required to complete either the uphill or downhill tests. These findings suggest that, in short and highly irregular uphill and downhill sections, 29-inch wheels do not necessarily improve performance over 26-inch wheels, although they modify the distribution of vibrations transmitted to the rider.

## 1. Introduction

Cross-country mountain biking (XC-MTB) is performed on uneven and irregular terrain, where rocks, bumps, steps, and other surface discontinuities expose riders to repeated impacts and vibrations. These mechanical disturbances can affect bicycle control, rider comfort, and performance, particularly in technical sections where maintaining balance and trajectory is essential [1]. Sudden impacts and irregularities may disrupt the rider’s balance and increase the risk of loss of control and accidents [2]. In addition, terrain-induced vibrations have been associated with discomfort, fatigue, and potential health-related consequences when exposure is repetitive or prolonged [3,4].

The transmission of vibration from the bicycle to the rider is particularly relevant at the hands, wrists, and upper limbs because these regions are exposed to continuous gripping forces and repeated impacts through the handlebars. Upper-extremity disorders in cyclists have been linked to high forces and prolonged gripping demands [5]. Prolonged pressure on the median and ulnar nerves, combined with jolts, impacts, and vibrations transmitted to the hands, has been associated with peripheral nerve symptoms and hand or wrist disorders [6,7,8]. Moreover, vibration-induced fatigue and reduced bicycle control may negatively influence performance during demanding off-road riding [9]. Recent evidence highlights that continuous exposure to steering-induced loads can significantly alter the vibrotactile perception threshold in cyclists’ upper limbs, exacerbating local discomfort and accelerating the onset of hand numbness [10]. Therefore, identifying equipment- and terrain-related factors that modify vibration transmission is important for improving comfort, reducing potential health risks, and optimizing performance.

Surface characteristics are among the main determinants of vibration exposure in cycling. In road cycling, rougher surfaces such as cobblestones produce higher effective acceleration values than smoother asphalt surfaces [11]. Comparisons between road and off-road cycling have also shown that vibrations are greater during off-road riding, both in the bicycle and at body–bicycle contact points [12,13]. However, vibrations measured at the head and lower back appear to be comparatively attenuated, suggesting that the rider’s musculoskeletal system plays an important role in absorbing mechanical disturbances before they reach more proximal body segments [13,14]. This attenuation may require additional non-propulsive muscular work, as riders use isometric contractions to stabilize the bicycle over rough terrain. Vibration exposure has also been related to temporary reductions in muscular recruitment and force production, which may increase physiological demand during off-road cycling [12,15,16].

Bicycle design has evolved substantially in recent decades to improve off-road performance, comfort, and control. Suspension systems, modified drivetrain components, hydraulic seat posts, changes in frame geometry, and larger wheel diameters are among the most relevant developments in XC-MTB equipment [17]. Front and full-suspension systems have generally been recommended for bumpy terrain because they can reduce vibration exposure and improve efficiency under certain riding conditions [18,19,20,21]. Tire characteristics are also relevant: larger tire volumes and lower inflation pressures may improve impact attenuation, comfort, and performance, provided that mechanical safety limits are not exceeded [14].

Wheel diameter has also received considerable attention. Although 26-inch wheels were historically standard in mountain biking, 27.5- and 29-inch wheels became widely adopted after their introduction to the market [1]. Manufacturers often claim that larger wheels improve obstacle roll-over, comfort, and performance. This is particularly relevant given that contemporary Olympic Cross-Country racing has evolved into circuits that are up to 40% shorter, highly explosive, and more technically demanding, which challenge the applicability of historical equipment paradigms under brief and intense efforts [22]. However, empirical findings remain mixed. Some studies suggest that larger wheel diameters may improve performance but also increase vibration transmission in cross-country conditions [1,17]. In contrast, other studies have reported that wheel size does not substantially affect performance, muscle activity, or tri-axial accelerations during MTB, suggesting that rider preference, sponsorship, and riding context may also influence wheel-size selection [23,24]. Therefore, the effects of wheel diameter on vibration transmission and performance remain insufficiently resolved.

Most previous studies have examined wheel-size effects during cross-country circuits or simulated competitive conditions. Although these protocols include rough sections, they do not always isolate the specific mechanical effect of repeated obstacles such as rocks or steps. Limited research has analyzed the effects of discrete obstacles on bicycle and rider vibration responses, with previous findings indicating that tire volume and inflation pressure can meaningfully alter vibration transmission and performance [13]. However, to the best of the authors’ knowledge, no study has directly compared equivalent 26- and 29-inch mountain bikes using an analytical field test specifically designed to emphasize the effect of repeated rocky steps during both uphill and downhill riding.

Therefore, the primary aim of this study was to analyze the effect of 26- versus 29-inch wheel diameter on vibrations recorded at different bicycle and rider locations during a short uphill and downhill test over rough, rocky terrain. A secondary aim was to examine whether wheel diameter affected performance, defined as the time required to complete each section. Based on previous research, we hypothesized that the 29-inch bicycle would produce higher vibration values at bicycle–rider contact points, whereas vibrations at the lower back and head would be lower than those recorded at the extremities and bicycle structure. We also hypothesized that the 29-inch bicycle would be faster than the 26-inch bicycle in both uphill and downhill sections.

## 2. Materials and Methods

### 2.1. Participants

Forty-nine highly trained and experienced male mountain bikers voluntarily participated in this study. Participants had a mean age of 35.90 ± 9.67 years, a mean height of 1.74 ± 0.04 m, and a mean body mass of 72.00 ± 8.63 kg. To reduce the influence of bicycle fit on vibration transmission, only cyclists with a height between 1.68 and 1.78 m were included, as both bicycles used in the study were medium-sized frames. Exclusion criteria were the use of medication that could affect neuromuscular function, the presence of any disease that could impair muscular or circulatory function, and any injury during the previous two years that could affect cycling performance or vibration response [25].

The study was approved by the Ethics Committee of the University of Valencia (code: H1493375377463) and was conducted in accordance with the Declaration of Helsinki. All participants were informed about the characteristics, potential benefits, and risks of the study before participation. Written informed consent was obtained from all cyclists, and participants were informed that they could withdraw from the study at any time.

### 2.2. Bicycles and Instruments

**Figure 1:**
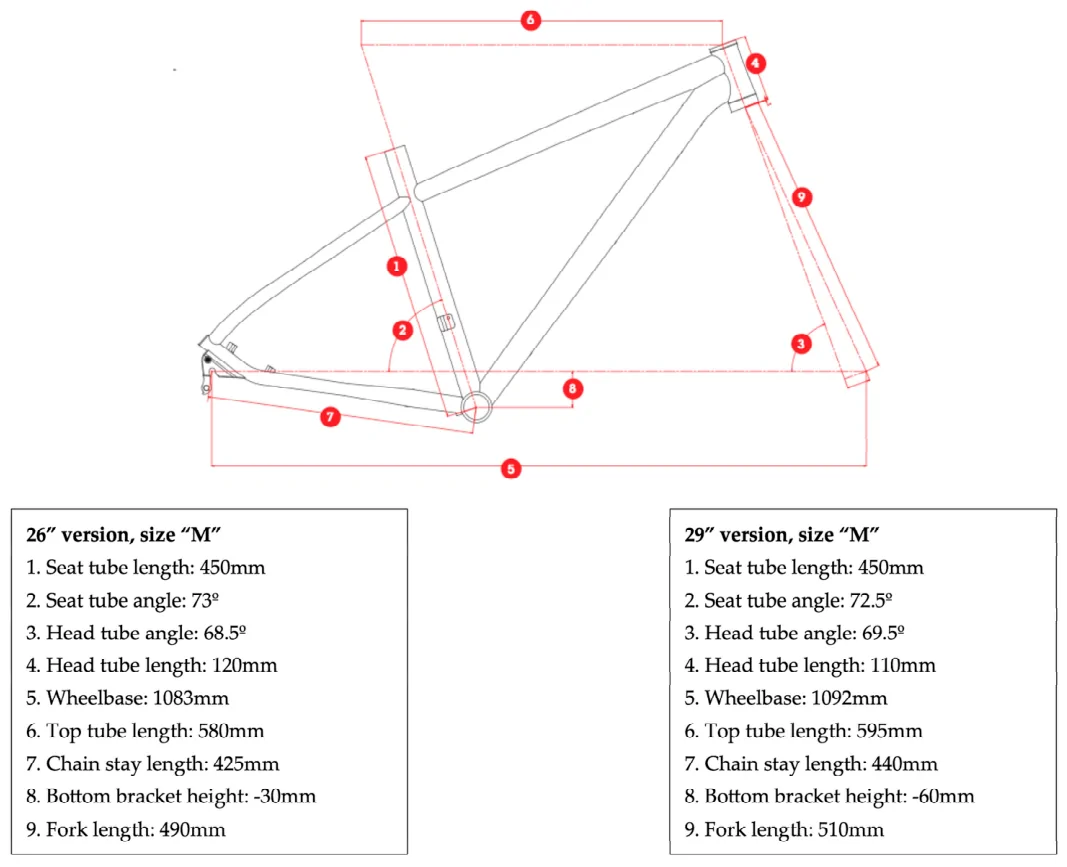
Comparison of the geometries of the 2013 Commencal Supernormal for the 26-inch and 29-inch versions.

Two Commencal Supernormal 2013 hardtail mountain bikes were used: one with 26-inch wheels and one with 29-inch wheels. Both bicycles had an NEC + ULTRA SL Aluminium 6061 triple-butted frame and equivalent components and setup. Figure 1 shows the full geometries and specifications of both bicycles.

The 26-inch bicycle had a mass of 10.80 kg, whereas the 29-inch bicycle had a mass of 11.50 kg. This 0.70 kg difference was expected because of the larger frame, wheels, and associated components of the 29-inch model. However, the difference was below the threshold reported by Berry et al. [26], who found that a 2 kg difference in off-road bicycle mass did not produce measurable differences in physiological variables such as oxygen uptake, heart rate, or ratings of perceived exertion.

These characteristics are directly linked to commercially available wheel sizes, establishing the baseline for this comparative study. Specifically, we assume that bicycles with larger wheels exhibit greater mass, lower acceleration, superior obstacle negotiation, and enhanced momentum retention. Conversely, 26-inch models are expected to be lighter, with higher acceleration, reduced obstacle-overcoming efficacy, and lower velocity maintenance. However, this research evaluates off-the-shelf bicycles in their original manufacturer configurations, precisely as purchased by consumers. Consequently, no component modifications were performed to standardize variables (such as adding ballast to the 26-inch model), thereby preserving the factory specifications and ecological validity of the equipment.

Both bicycles were adjusted to match the participants’ anthropometric characteristics as closely as possible. Shimano pedals were used so that all participants could ride with their own Shimano Pedaling Dynamics (SPD)-compatible cycling shoes. Tires were Onza Canis Skinwall 26 × 2.25 for the 26-inch bicycle and 29 × 2.25 for the 29-inch bicycle. Tire pressure was set at 2 bar for both bicycles, following the manufacturer’s recommendations. No additional changes were made to the bicycles during the testing session.

Vibrations were measured using eight triaxial ActiGraph accelerometers (ActiGraph Corp., wGT3X-BT, Pensacola, FL, USA). The accelerometers were programmed to record acceleration in the three spatial axes at a sampling frequency of 100 Hz. This frequency was selected because previous off-road cycling research has shown that the main excitations at the bicycle–body interface occur below 50 Hz [6,18,25,27]. Two accelerometers were attached to the bicycle, with one on the front hub (FH) and one on the rear hub (RH). Six accelerometers were placed on the rider: helmet (HE), coccyx (CO), left wrist (LW), right wrist (RW), left ankle (LA), and right ankle (RA). These locations were selected according to previous research on vibration transmission in mountain biking [17].

Performance was assessed as the time required to complete each section of the test. Timing was recorded using a pair of electronic photocells installed at the start and finish of the track, with their corresponding reflectors (Chronojump Bosco System, Chronojump Photocell, precision 1 ms, 12–220 V DC/AC). Photocell data were acquired using Chronojump software 1.5.3 installed on a laptop positioned at the end of the route.

### 2.3. Experimental Protocol

Participants were instructed to maintain their usual diet and to avoid strenuous exercise during the 48 h before the test session [28]. Before testing, the accelerometers were attached to the bicycle and rider, and correct placement and signal recording were checked. Participants then completed a familiarization lap on the track to warm up and to become accustomed to the course and testing procedure.

Each participant completed six trials in total: three with the 26-inch bicycle and three with the 29-inch bicycle. Bicycle order was randomized to reduce potential learning, fatigue, or order effects [29]. Participants were instructed to complete each trial in the shortest possible time while remaining within the marked track. Each trial consisted of an uphill section followed by a downhill section. The uphill section was performed pedaling, whereas the downhill section was performed without pedaling to reduce the influence of active propulsion and to focus on vibration transmission during freewheeling over the obstacles.

Before the start of each trial, a researcher assisted the cyclist in maintaining a stable starting position by holding the rear wheel between his legs and gripping the seatpost, while the rider adopted a standing time-trial position. Because of the steepness, surface irregularities, and characteristics of the track, riding while seated was not feasible. Therefore, the entire test was completed standing on the pedals. After reaching the top of the track, participants turned around and prepared for the downhill section, adopting the same assisted starting position as in the uphill section.

Following the uphill section, participants rested for 60 s to recover from fatigue and position their bicycles for the downhill. After the descent, the cyclists completed a 3 min recovery period prior to the next repetition (i.e., 6 in total, alternating order).

For each bicycle and section, the three repeated trials were averaged for each participant before statistical analysis. This procedure reduced trial-to-trial variability and ensured that each participant contributed one representative value per bicycle and condition to the repeated-measures analyses.

### 2.4. Field Test

**Figure 2:**
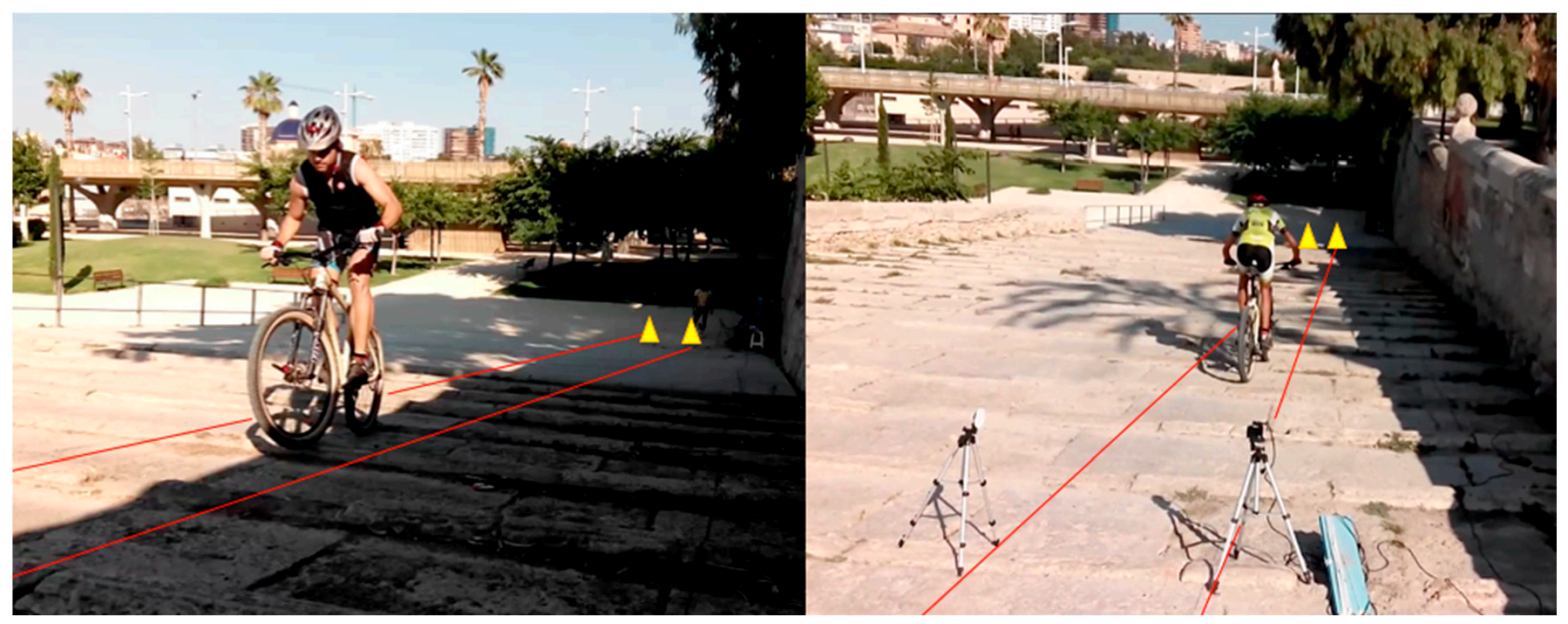
Field test setup. (**Left**) participant performing the uphill test. (**Right**) participant performing the downhill test. The red lines indicate the ropes used to delimit the course, and the yellow markers indicate the position of the photocells.

The field test was conducted on a 52.3 m trail located on an old stone jetty. The course included a bumpy uphill and downhill section with a 16% gradient. The track contained 28 repeated steps with a median height of 0.08 m, a surface length of 0.26 m, rounded edges, and a perpendicular orientation to the direction of travel. The steps were distributed at intervals of 0.95 m. The lowest section of the ramp, measuring 15.73 m, did not contain steps, allowing participants to accelerate before reaching the stepped section during the uphill test. The complete execution of the field tests can be observed in the Supplementary Video S1 and the field test setup is shown in Figure 2.

### 2.5. Data Processing

Acceleration signals were processed using MATLAB 2015 (MathWorks Inc., Natick, MA, USA), following procedures used in previous mountain biking vibration studies [1,17]. First, the resultant acceleration vector was calculated from the three orthogonal axes of each accelerometer. This procedure was used because accelerometer orientation could vary slightly during field testing, especially at body-mounted locations. This triaxial vector resultant approach is consistent with contemporary dynamic comfort mapping methodologies that assess multi-axis structural and biological accelerations to isolate mechanical disturbances regardless of sensor orientation changes [30].

To remove low-frequency noise and gravitational components, the resultant acceleration signals were high-pass-filtered using a cutoff frequency of 0.25 Hz [31]. The root mean square (RMS) of the filtered resultant acceleration was then calculated for each accelerometer location and trial. RMS acceleration was used as the primary indicator of vibration magnitude.

To assess bicycle-to-rider vibration transmission, RMS ratios were calculated between selected body locations and bicycle reference points. The following ratios were computed: left wrist/front hub (LW/FH), right wrist/front hub (RW/FH), left ankle/rear hub (LA/RH), right ankle/rear hub (RA/RH), coccyx/rear hub (CO/RH), and helmet/front hub (HE/FH). These ratios were used to describe the relative transmission of vibration from the bicycle structure to the rider at the main contact or proximal body locations.

### 2.6. Statistical Analysis

Statistical analyses were performed using GraphPad Prism Version 9 (GraphPad Software, San Diego, CA, USA). Data from two participants were excluded from the acceleration analyses because of accelerometer signal loss. Therefore, the vibration analyses were performed with 47 participants, whereas the performance analyses were performed with the complete sample of 49 participants.

The normality of the dependent variables was visually inspected and assessed before inferential analysis. For performance, a two-way repeated-measures analysis of variance (ANOVA) was used with wheel diameter (26-inch and 29-inch) and slope section (uphill and downhill) as within-subject factors. For RMS acceleration, two separate two-way repeated-measures ANOVAs were conducted for the uphill and downhill sections, with wheel diameter (26-inch and 29-inch) and accelerometer location (helmet, coccyx, front hub, rear hub, left wrist, right wrist, left ankle, and right ankle) as within-subject factors.

When significant main effects or interactions were detected, post hoc pairwise comparisons were performed using Tukey’s multiple-comparison procedure. When the assumption of sphericity was violated, the Greenhouse–Geisser correction was applied, and corrected degrees of freedom were reported. Vibration transmission ratios were compared between the 26- and 29-inch bicycles using paired-samples *t*-tests. Effect sizes were calculated using partial eta squared (ηp^2^) for ANOVA effects and Cohen’s d for paired comparisons. The significance level was set at *p* < 0.05 for all analyses.

## 3. Procedure

### 3.1. Effect of Accelerometer Location and Wheel Diameter on RMS Acceleration

**Figure 3:**
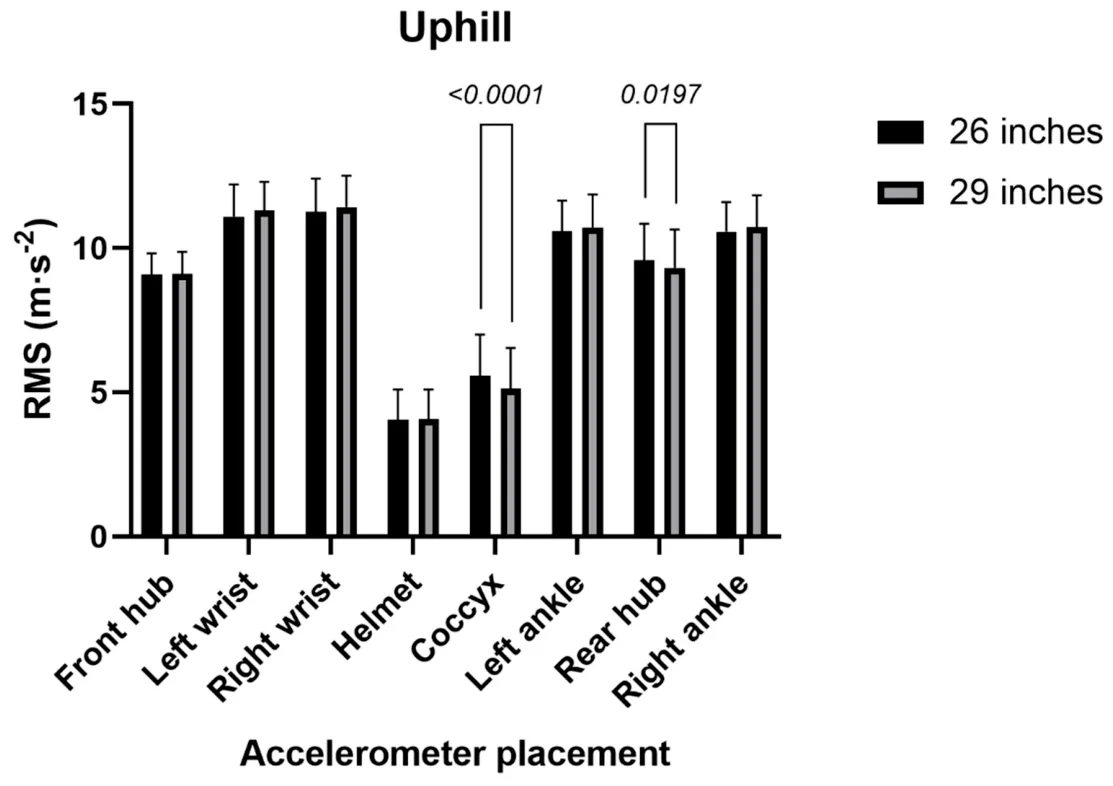
RMS acceleration during the uphill test according to wheel diameter and accelerometer location. Values are presented for the 26- and 29-inch bicycles at the front hub, left wrist, right wrist, helmet, coccyx, left ankle, rear hub, and right ankle. Brackets indicate significant pairwise differences between wheel diameters at the same accelerometer location.

Acceleration data from two participants were excluded because of signal loss. Therefore, RMS acceleration analyses were performed with 47 participants. During the uphill section, accelerometer location had a significant effect on RMS acceleration, F(2.381, 109.5) = 556.6, *p* < 0.001, ηp^2^ = 0.92. A significant accelerometer location–wheel diameter interaction was also observed, F(3.982, 183.2) = 16.25, *p* < 0.001, ηp^2^ = 0.26. Pairwise comparisons showed that the 26-inch bicycle produced significantly higher RMS acceleration values than the 29-inch bicycle at the coccyx (*p* < 0.0001) and rear hub (*p* = 0.0197). No significant differences between wheel diameters were observed at the remaining accelerometer locations during the uphill section (Figure 3).

**Figure 4:**
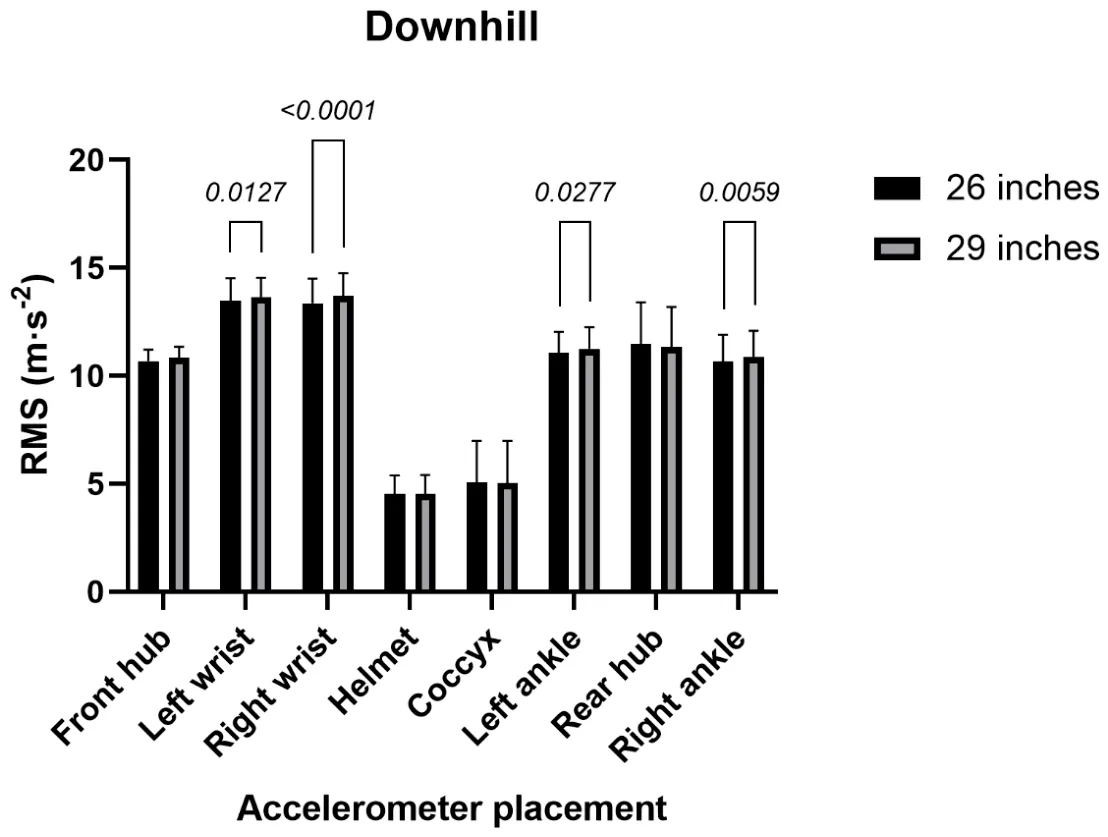
RMS acceleration during the downhill test according to wheel diameter and accelerometer location. Values are presented for the 26- and 29-inch bicycles at the front hub, left wrist, right wrist, helmet, coccyx, left ankle, rear hub, and right ankle. Brackets indicate significant pairwise differences between wheel diameters at the same accelerometer location.

During the downhill section, accelerometer location had a significant effect on RMS acceleration, F(7, 322) = 382.0, *p* < 0.001, ηp^2^ = 0.89. A significant main effect of wheel diameter was also found, F(1, 46) = 14.58, *p* = 0.004, ηp^2^ = 0.24, together with a significant accelerometer location–wheel diameter interaction, F(7, 322) = 10.14, *p* < 0.001, ηp^2^ = 0.18. Pairwise comparisons indicated that the 29-inch bicycle produced significantly higher RMS acceleration values than the 26-inch bicycle at the left wrist (*p* = 0.0127), right wrist (*p* < 0.0001), left ankle (*p* = 0.0277), and right ankle (*p* = 0.0059). No significant differences between wheel diameters were observed at the front hub, helmet, coccyx, or rear hub during the downhill section (Figure 4).

### 3.2. Effect of Wheel Diameter on Vibration Transmission

**Figure 5:**
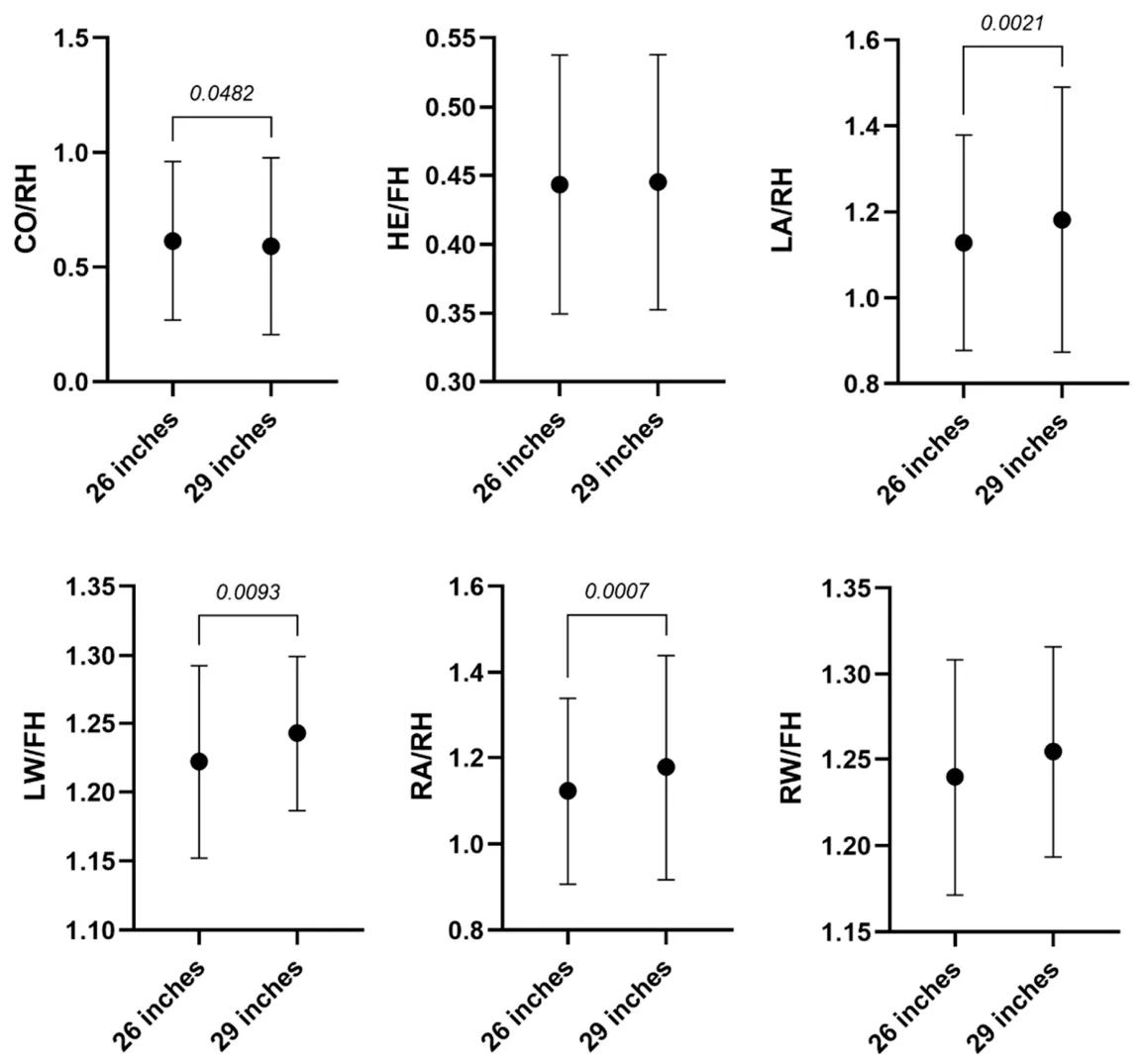
Vibration transmission ratios during the uphill test according to wheel diameter. Ratios were calculated between selected rider locations and bicycle reference points: coccyx/rear hub (CO/RH), helmet/front hub (HE/FH), left ankle/rear hub (LA/RH), left wrist/front hub (LW/FH), right ankle/rear hub (RA/RH), and right wrist/front hub (RW/FH). Brackets indicate significant pairwise differences between wheel diameters.

During the uphill section, significant differences between wheel diameters were found in four of the six vibration transmission ratios analyzed. The 29-inch bicycle showed higher transmission ratios than the 26-inch bicycle for left wrist/front hub, t(46) = 2.71, *p* = 0.009, d = 0.80; left ankle/rear hub, t(46) = 3.26, *p* = 0.002, d = 0.96; and right ankle/rear hub, t(46) = 3.64, *p* < 0.001, d = 1.07. In contrast, the 26-inch bicycle showed a higher coccyx/rear hub ratio than the 29-inch bicycle, t(46) = 2.03, *p* = 0.048, d = 0.60. No significant differences were observed for helmet/front hub or right wrist/front hub ratios (Figure 5).

**Figure 6:**
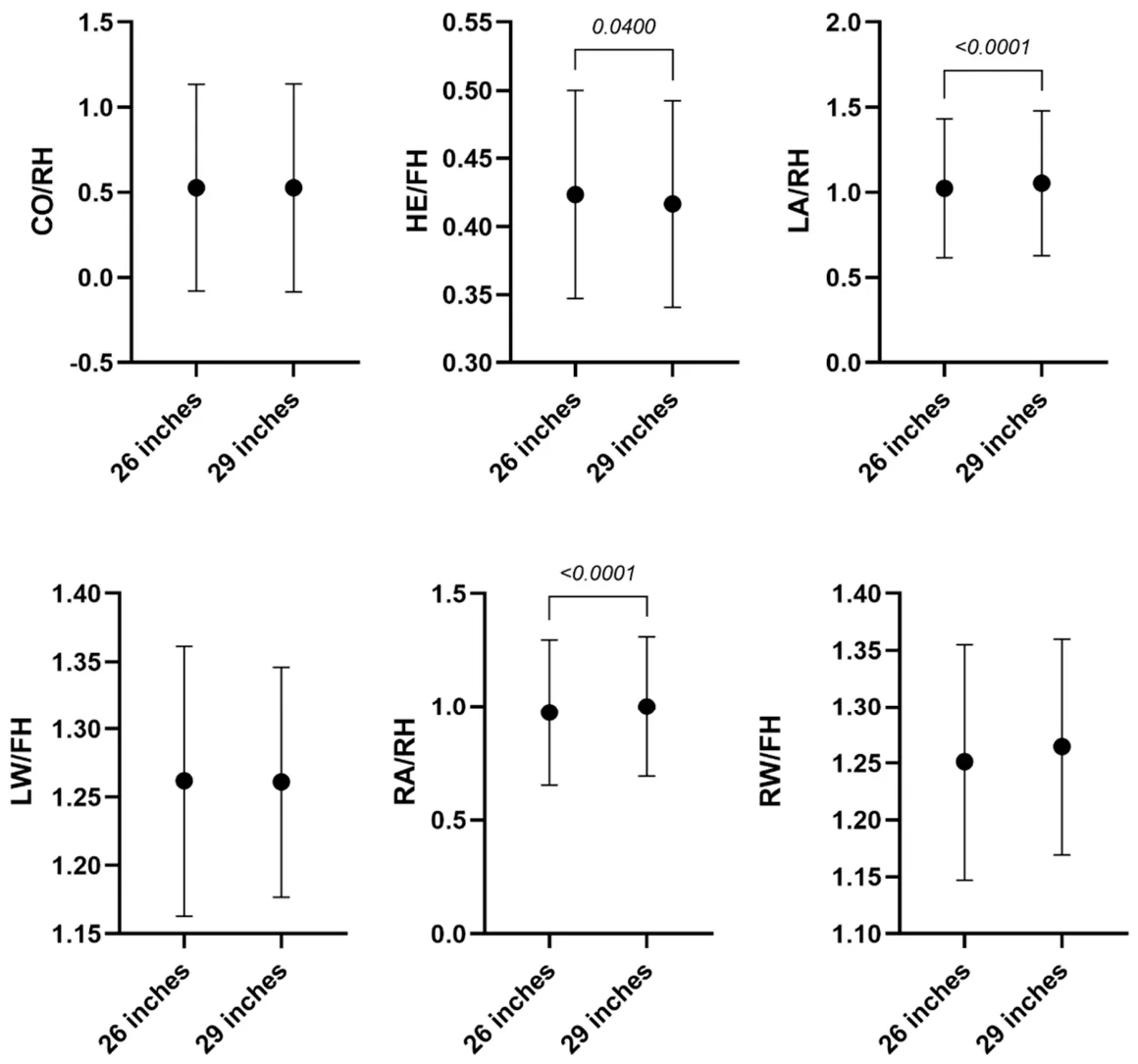
Vibration transmission ratios during the downhill test according to wheel diameter. Ratios were calculated between selected rider locations and bicycle reference points: coccyx/rear hub (CO/RH), helmet/front hub (HE/FH), left ankle/rear hub (LA/RH), left wrist/front hub (LW/FH), right ankle/rear hub (RA/RH), and right wrist/front hub (RW/FH). Brackets indicate significant pairwise differences between wheel diameters.

During the downhill section, significant differences between wheel diameters were observed in three vibration transmission ratios. The 29-inch bicycle showed higher transmission ratios than the 26-inch bicycle for left ankle/rear hub, t(46) = 4.69, *p* < 0.001, d = 1.38, and right ankle/rear hub, t(46) = 4.48, *p* < 0.001, d = 1.32. Conversely, the 26-inch bicycle showed a higher helmet/front hub ratio than the 29-inch bicycle, t(46) = 2.11, *p* = 0.040, d = 0.62. No significant differences were observed for coccyx/rear hub, left wrist/front hub, or right wrist/front hub ratios (Figure 6).

### 3.3. Performance

**Figure 7:**
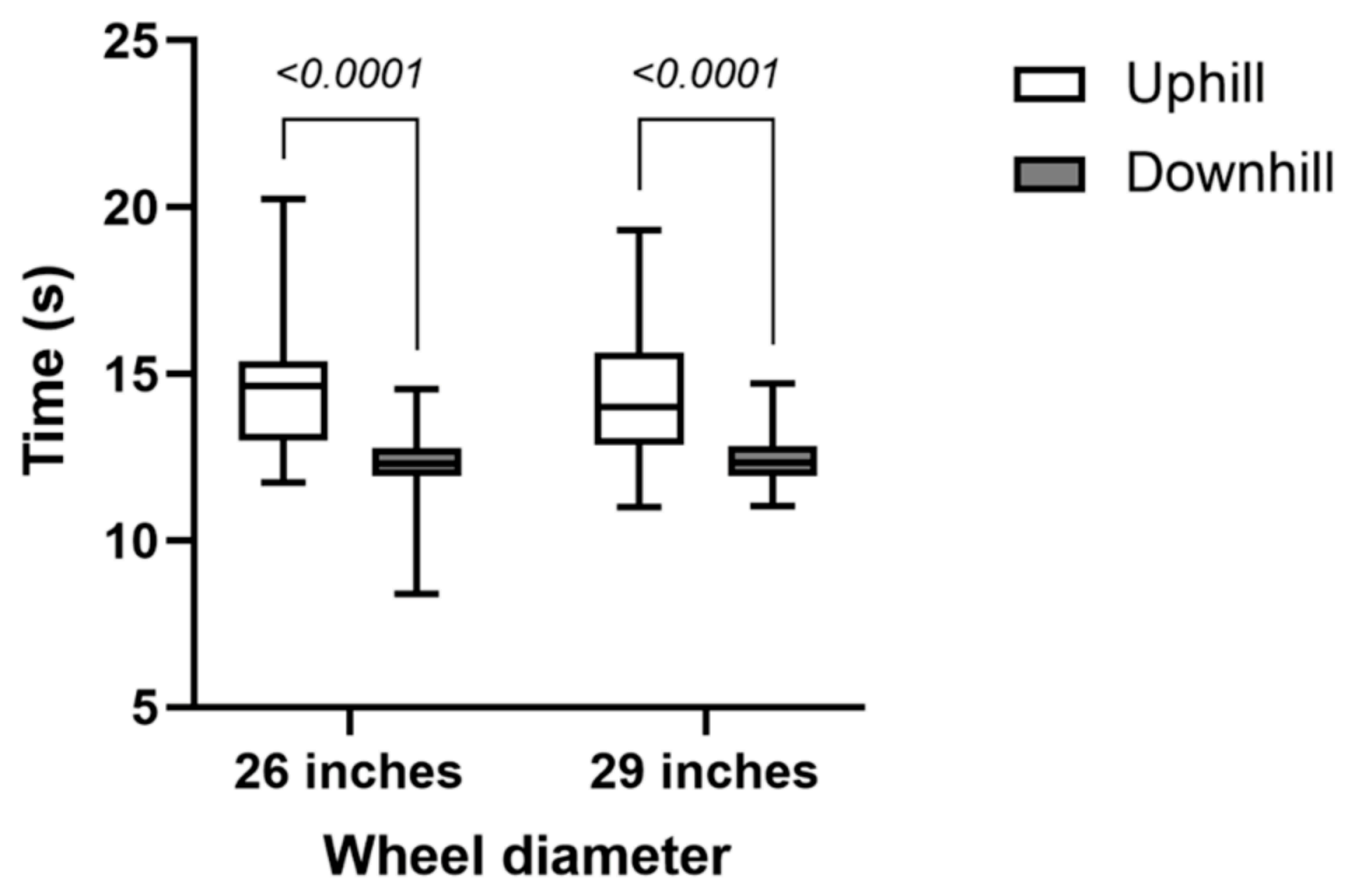
Time required to complete the uphill and downhill tests according to wheel diameter. Values are shown for the 26- and 29-inch bicycles. Brackets indicate significant differences between uphill and downhill sections within each wheel diameter.

Performance was analyzed as the time required to complete the uphill and downhill sections. A significant main effect of slope section was found, F(1, 48) = 71.54, *p* < 0.001, ηp^2^ = 0.59. Participants completed the downhill section faster than the uphill section with both bicycles. However, no significant differences were observed between the 26- and 29-inch bicycles in the time required to complete either the uphill or downhill section (*p* > 0.05). These results indicate that wheel diameter did not significantly affect performance in this specific short uphill and downhill field test (Figure 7).

## 4. Discussion

This study compared vibration transmission and performance between equivalent 26- and 29-inch hardtail mountain bikes during a short uphill and downhill field test over rough, rocky terrain. The main findings were that wheel diameter significantly affected RMS acceleration and bicycle-to-rider vibration transmission, whereas it did not significantly affect the time required to complete either the uphill or downhill section. Therefore, the hypothesis that the 29-inch bicycle would be faster than the 26-inch bicycle in this specific task was not supported. However, the hypothesis that wheel diameter would modify vibration responses was partially supported, as the differences between bicycles depended on the slope section and accelerometer location.

During the uphill section, the 26-inch bicycle produced higher RMS acceleration values at the coccyx and rear hub than the 29-inch bicycle. This result may be related to the interaction between obstacle geometry and wheel diameter. Larger wheels are generally considered to improve obstacle rollover because they reduce the angle of attack when contacting irregularities [1,17]. In the present uphill test, this mechanical advantage may have reduced the vertical impact trans-mitted through the rear part of the bicycle and toward the lower back. However, no significant differences between wheel diameters were found at the remaining accelerometer locations during the uphill section, suggesting that the effect of wheel diameter was not uniform across the bicycle–rider system.

During the downhill section, the pattern differed. The 29-inch bicycle produced higher RMS acceleration values at the wrists and ankles, whereas no significant differences were observed at the front hub, rear hub, helmet, or coccyx. Because the downhill section was performed without pedaling, these differences cannot be attributed to differences in active propulsion. Instead, they suggest that wheel diameter may influence how vibrations are distributed through the rider’s extremities during freewheeling over repeated rocky steps. This finding is consistent with previous research showing that larger wheel diameters may be associated with increased vibration transmission in some cross-country mountain biking conditions [1,17]. Nevertheless, the present results also indicate that greater vibration at the extremities does not necessarily imply greater vibration at more proximal body segments such as the lower back or head.

From a practical and clinical perspective, although these statistically significant vibration differences did not alter short-term performance times, their cumulative effects during a full-length cross-country race could be meaningful for competitive riders [1,3]. The increased vibration transmission to the wrists and ankles observed with 29-inch wheels during downhills may accelerate localized neuromuscular fatigue and compound mechanical stress on peripheral nerves, potentially elevating the risk of upper-limb compression disorders such as handlebar palsy during prolonged exposure [5,32]. Conversely, the higher accelerations at the coccyx and rear hub for the 26-inch bicycle during uphills suggest that riders face a greater mechanical burden at the lower back. This requires additional non-propulsive isometric muscle work to stabilize the torso, which can increase physiological demand and negatively impact long-term riding economy over an extended duration [12]. For elite competitive athletes, where performance is optimized through precise fatigue management, these subtle shifts in vibration distribution mean that wheel-size selection involves a distinct trade-off between localized upper-extremity and lower-back physical strain [17].

The vibration transmission ratios provide additional insight into this pattern. During the uphill section, the 29-inch bicycle showed higher transmission ratios at the left wrist and both ankles, whereas the 26-inch bicycle showed a higher coccyx/rear hub ratio. During the downhill section, the 29-inch bicycle showed higher transmission ratios at both ankles, whereas the 26-inch bicycle showed a higher helmet/front hub ratio. Additionally, while hand-arm vibration has been widely characterized, recent evidence highlights that foot-transmitted vibration during cycling represents an essential yet understudied component of total mechanical exposure [33], aligning with the high values observed at the ankles in the current protocol. These results suggest that the 29-inch bicycle may transmit relatively more vibration to the extremities, while vibration reaching the helmet and coccyx may be attenuated differently depending on the section and contact point. Previous studies have suggested that the rider’s musculoskeletal system can attenuate vibrations before they reach the head and lower back [13,14]. The present findings are compatible with that interpretation, as the helmet and coccyx generally showed lower vibration values than the wrists, ankles, and bicycle hubs.

The lower vibration values observed at the helmet and coccyx are also relevant from a biomechanical perspective. Off-road cycling requires riders to stabilize the bicycle and absorb repeated impacts through the upper and lower limbs. This stabilization may involve non-propulsive muscular work and isometric contractions, especially when riding over rough terrain [12]. Vibration exposure has also been associated with temporary reductions in muscular recruitment and force production, which may contribute to fatigue during demanding cycling tasks [15,16]. Therefore, the fact that the highest vibration values were recorded at the wrists and ankles is consistent with the role of the extremities as primary interfaces for absorbing and controlling impacts during mountain biking. Furthermore, recent field characterizations using inertial measurement units confirm that lower extremities exhibit distinct localized multi-axis acceleration spikes linked to surface irregularities and pedaling phase conditions, reinforcing the role of ankles and feet as critical dampening interfaces [33].

No significant performance differences were found between the 26- and 29-inch bicycles in either the uphill or downhill section. This finding contrasts with the expectation that the 29-inch bicycle would be faster because of its greater obstacle rollover capacity, as suggested in previous research on cross-country riding [1,17]. However, it is also consistent with studies reporting that wheel size does not necessarily produce clear performance advantages in mountain biking [23,24]. In the present study, the short duration, steep gradient, standing position, and repeated rocky steps may have reduced or neutralized any potential performance advantage of the 29-inch wheels. The greater rollover capacity of the 29-inch bicycle may have been counterbalanced by other factors, such as its slightly greater mass, different frame geometry, and lower acceleration capacity in a short uphill effort.

The absence of performance differences is particularly relevant because it indicates that greater vibration transmission at some body locations did not translate into faster completion times. In other words, the 29-inch bicycle modified the distribution of vibrations but did not improve performance in this specific task. This supports the idea that wheel-size selection should not be based only on general assumptions about larger wheels being faster or more efficient. Instead, the effect of wheel diameter appears to depend on the characteristics of the terrain, the length and slope of the section, the type of obstacles, and the demands placed on the rider.

From an applied perspective, these findings suggest that 29-inch wheels should not automatically be assumed to provide a performance advantage in short, highly irregular uphill and downhill sections. Although larger wheels may improve obstacle rollover in some contexts, they may also increase vibration transmission to the wrists and ankles. Therefore, equipment selection should consider not only performance but also comfort, vibration exposure, rider morphology, and the specific characteristics of the terrain. Tire volume and inflation pressure may also be important variables, as previous research has shown that larger tire volumes and lower pressures can improve impact attenuation and performance when used within safe mechanical limits [12]. In this sense, wheel diameter should be considered together with tire characteristics, suspension configuration, and bicycle geometry rather than as an isolated factor. This holistic view is supported by recent experimental data showing a non-linear interaction where the physical properties of the wheel assembly alter frame-transmitted vibrations depending on the terrain roughness and tire compliance parameters [34].

Several limitations should be acknowledged. First, although the bicycles were equivalent in components and setup, they were not identical in all mechanical characteristics. The 29-inch bicycle was slightly heavier and necessarily differed in wheel size, frame dimensions, and geometry. Therefore, the observed effects should be interpreted as the result of the complete bicycle configuration associated with each wheel size, rather than as the isolated effect of wheel diameter alone. Second, the sample included only highly trained male mountain bikers within a restricted height range, which improves experimental control but limits generalization to female cyclists, recreational riders, children, or cyclists with different anthropometric characteristics. Third, the field test was intentionally short and highly specific, with repeated rocky steps and a 16% gradient. This design was useful for emphasizing vibration responses, but it does not represent the full complexity of cross-country mountain biking races or longer recreational rides. Fourth, rider posture, joint kinematics, muscle activity, perceived comfort, and perceived stability were not measured. Therefore, explanations related to body position, muscular attenuation, or rider confidence should be considered plausible interpretations rather than direct findings.

Future studies should examine wheel-size effects in longer and more varied mountain biking conditions, including different gradients, obstacle types, tire pressures, tire volumes, and suspension configurations. This study evaluated only one bicycle model and component configuration, which may limit generalizability to modern full-suspension bicycles or other frame designs commonly used in contemporary cross-country mountain biking. The inclusion of electromyography, kinematic analysis, and subjective comfort or control ratings would also help clarify how riders actively modulate vibration transmission through posture and muscle activation. In addition, future research should include female cyclists, different skill levels, and a wider range of body sizes to determine whether the effects observed in the present study are consistent across different rider populations.

## 5. Conclusions

Wheel diameter influenced vibration magnitude and vibration transmission during a short uphill and downhill mountain biking test over rough, rocky terrain, but it did not significantly affect performance. Both bicycles showed a similar overall vibration pattern, with the lowest RMS acceleration values recorded at the helmet and coccyx and the highest values recorded at the wrists and ankles.

During the uphill section, the 26-inch bicycle produced higher vibration values than the 29-inch bicycle at the coccyx and rear hub. In contrast, during the downhill section, the 29-inch bicycle produced higher vibration values than the 26-inch bicycle at the wrists and ankles. Despite these differences in vibration behavior, no significant differences were found between wheel diameters in the time required to complete either the uphill or downhill section.

These findings suggest that 29-inch wheels should not automatically be assumed to provide a performance advantage over 26-inch wheels in short, highly irregular uphill and downhill sections. Instead, wheel diameter appears to modify how vibrations are distributed across the bicycle–rider system, which may have practical implications for equipment selection, comfort, and vibration exposure in mountain biking.

## Data Availability

The raw data supporting the conclusions of this article will be made available by the authors on request.

## Supplementary Materials

A video of the complete test is available at https://zenodo.org/records/15882317 (accessed on 11 August 2026).
